# Good and sustained response to pembrolizumab and pazopanib in advanced undifferentiated pleomorphic sarcoma: a case report

**DOI:** 10.1186/s13569-020-00133-9

**Published:** 2020-07-09

**Authors:** Shalabh Arora, Sameer Rastogi, Shamim Ahmed Shamim, Adarsh Barwad, Maansi Sethi

**Affiliations:** 1 Department of Medical Oncology, Dr B.R.A. Institute Rotary Cancer Hospital, All India Institute of Medical Sciences, New Delhi, India; 2 Department of Nuclear Medicine, All India Institute of Medical Sciences, New Delhi, India; 3 Department of Pathology, All India Institute of Medical Sciences, New Delhi, India; 4 Department of Ophthalmology, Lady Hardinge Medical College, New Delhi, India

**Keywords:** Pembrolizumab, Undifferentiated pleomorphic sarcoma, Pazopanib, Immunotherapy

## Abstract

**Background:**

Conventional cytotoxic agents and pazopanib are approved for advanced soft tissue sarcomas but have low response rates and modest survival benefits. Recently, immune checkpoint inhibitors have shown clinically meaningful activity. The combination of pazopanib and immunotherapy has shown synergism in various other malignancies but has not been fully explored in advanced soft tissue sarcomas.

**Case presentation:**

A 63 year old woman with metastatic undifferentiated pleomorphic sarcoma progressed after two lines of palliative combination chemotherapy—doxorubicin with olaratumab, and gemcitabine with docetaxel. In view of significant symptoms, she was treated with pazopanib in combination with pembrolizumab. She had remarkable radiological and clinical improvement, with a manageable toxicity profile and an ongoing response at ten months of therapy.

**Conclusions:**

Undifferentiated pleomorphic sarcoma is an immunologically active subtype of soft tissue sarcoma, which is particularly amenable to immune checkpoint inhibitors. Pazopanib with immune checkpoint inhibitors is a well-tolerated, yet hitherto underexplored combination that may offer significant clinical benefit in advanced sarcomas—this finding warrants further evaluation in clinical trials.

## Background

The outcomes in metastatic soft tissue sarcoma (mSTS) remain dismal even though various drugs have been added in treatment arsenal during this decade. Conventional cytotoxic agents like doxorubicin, ifosfamide and gemcitabine/docetaxel have modest activity and significant toxicities associated with their use. Pazopanib was the first targeted therapy that broke the dormancy in the landscape of mSTS based upon PALETTE trial and was approved by (US FDA) United States Food and Drug Administration in second line in non-adipocytic STS [1]. Subsequently trabectedin and eribulin were approved in second line in L-sarcomas (liposarcoma and leiomyosarcoma). This was followed by accelerated approval for olaratumab in first line after it showed unprecedented improvement in overall survival of 11.8 months in a small phase 2 trial [2]. However, the ANNOUNCE trial presented recently in American Society of Clinical Oncology (ASCO) 2019 meeting in abstract form showed lack of benefit and thereafter its FDA approval has been revoked [3].

Immune checkpoint inhibitors have shown promising results in many other tumors apart from sarcoma (melanoma, renal cell carcinoma, non-small cell lung cancer, Hodgkin’s lymphoma etc.) and are thus being explored in advanced STS. A multicenter phase 2 trial (SARC-028) evaluating pembrolizumab in advanced STS showed an overall response rate of 40% (4/10) in patients with undifferentiated pleomorphic sarcoma (UPS) but was ineffective in leiomyosarcoma (0/10) and moderately effective in liposarcoma (2/10) [4]. Subsequently George et al. showed the ineffectiveness of nivolumab in uterine leiomyosarcoma (LMS) [5]. The PEMBROSARC trial tested pembrolizumab in combination with metronomic cyclophosphamide for patients with LMS, UPS and other sarcomas [6]. None of the sixteen UPS patients in this report had a response to pembrolizumab.

Based upon the available data (which show somewhat conflicting results), liposarcoma and undifferentiated pleomorphic sarcoma are probably the sarcomas in which immunotherapy should be explored. Herein we present the case of a 63 year old patient with metastatic undifferentiated pleomorphic sarcoma who failed two lines of therapy but had a remarkable response with anti-programmed death protein-1 (anti-PD-1) antibody pembrolizumab in combination with the multitargeted small molecule tyrosine kinase inhibitor pazopanib.

## Case presentation

**Fig. 1:**
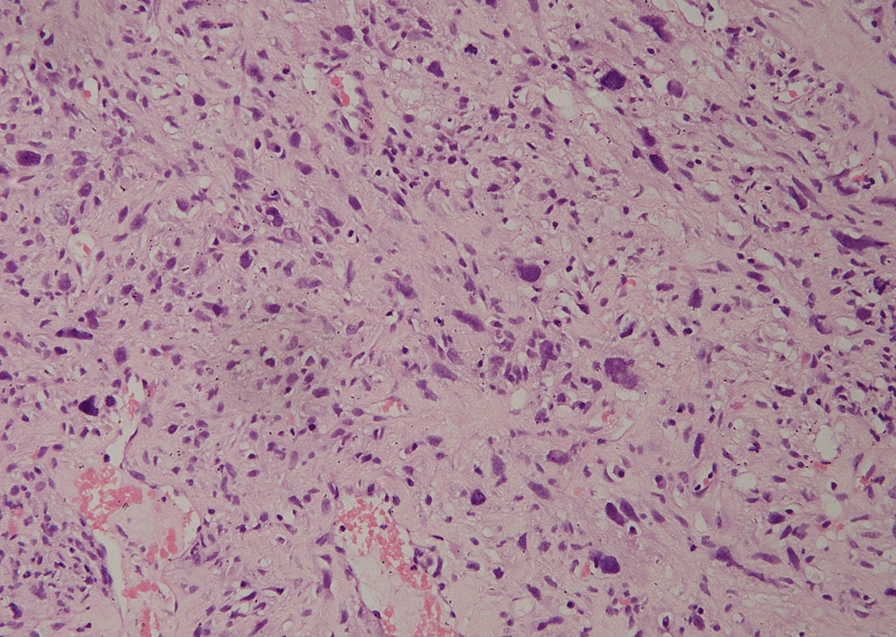
Histology photomicrograph of the excised right thigh soft tissue mass showing a malignant mesenchymal tumor with markedly pleomorphic spindle to bizarre cells exhibiting marked nuclear pleomorphism, coarse chromatin and abundant eosinophilic cytoplasm (H&E, 200x)

**Fig. 2:**
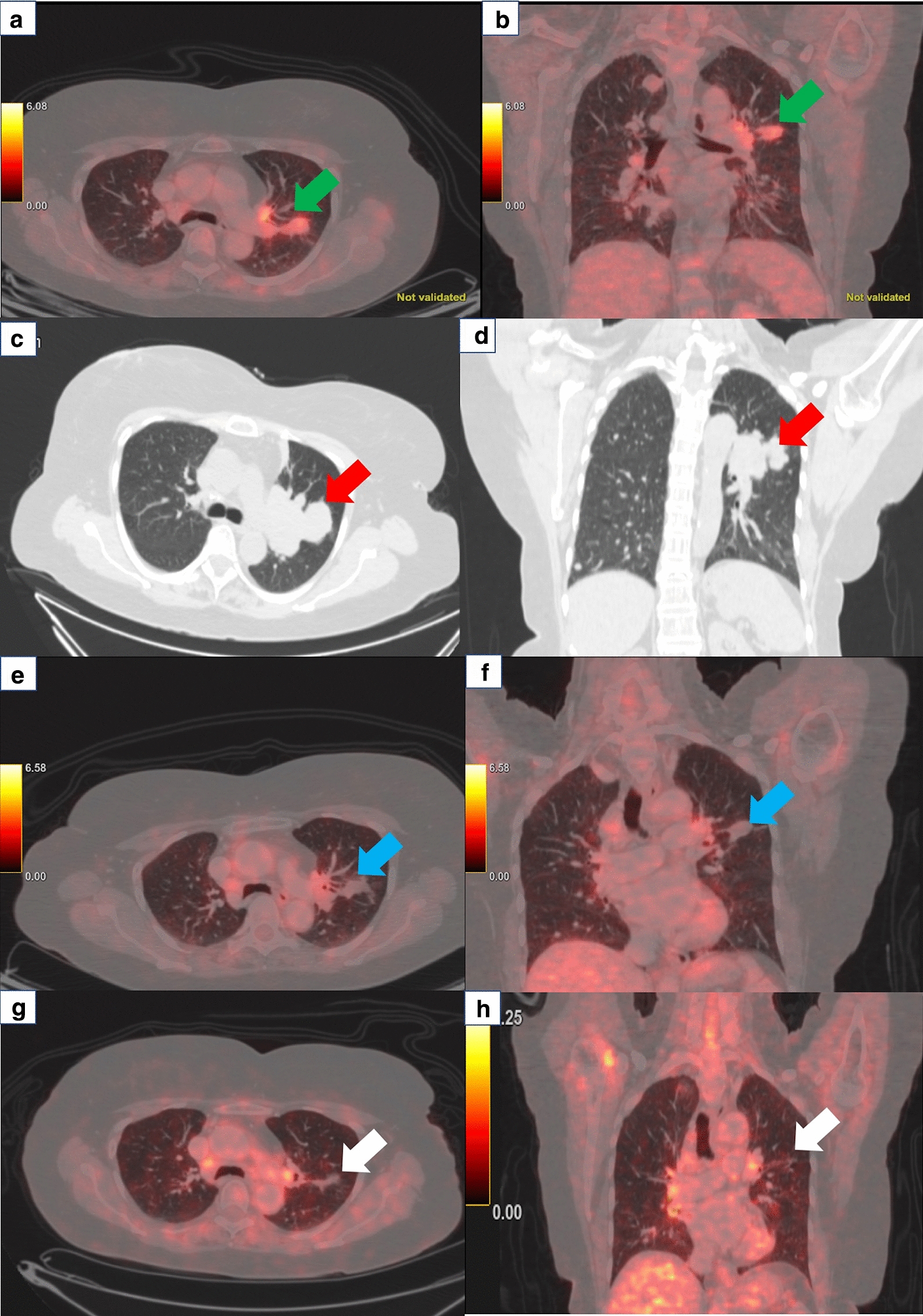
Axial (**a**) and coronal (**b**) fused positron emission tomography—computed tomography (PET-CT) images showing 18-fluorodeoxyglucose (18-FDG) avid soft tissue nodule in the left lung upper lobe (green arrows) after completion of gemcitabine-docetaxel chemotherapy. CT scan images (**c**, **d**) in lung window showing progression of nodules in left lung upper lobe (red arrows) after 3 months of watchful waiting. 18-FDG PET-CT scan images showing reduction in size with resolution of metabolic activity of lung nodules after 3 months (**e**, **f**) (blue arrows) and 9 months (**g**, **h**) (white arrows) of treatment with pembrolizumab + pazopanib

**Fig. 3:**
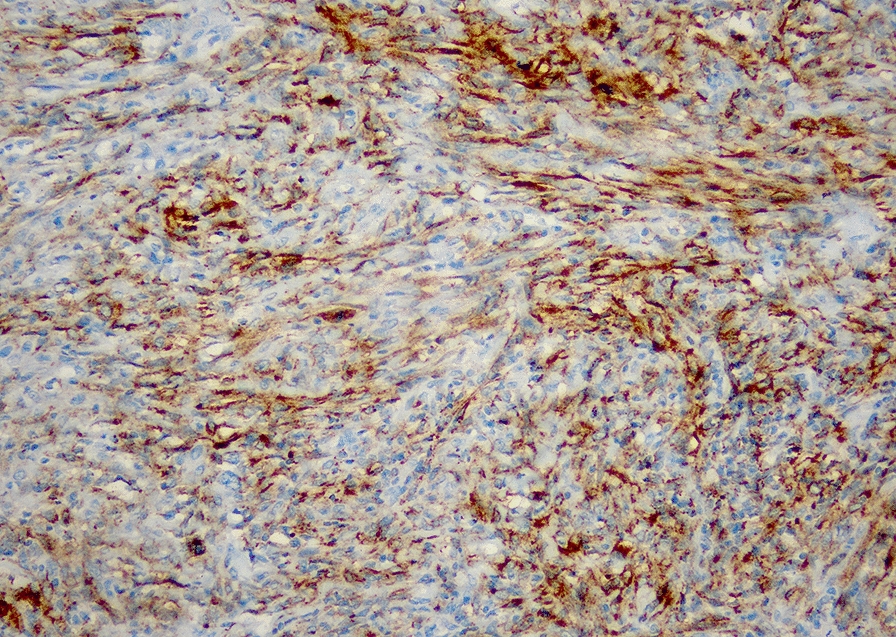
Immunohistochemistry for anti-programmed death ligand-1 antibody showing membranous positivity in tumor cells (Ventana SP263 assay)

A 63 year old woman with no known comorbidities, was evaluated in September 2017 for complaints of an insidious onset, gradually progressive painless swelling in the posterior aspect of right thigh. Magnetic resonance imaging scan revealed a well-defined, lobulated soft tissue lesion in posterior subcutaneous compartment of the right knee joint. She underwent excision biopsy of the primary lesion at a local hospital and histopathology was suggestive of undifferentiated pleomorphic sarcoma, with 14–15 mitoses per high power field, no necrosis and FNCLCC grade II (Fig. 1). Subsequently whole body 18-fluorodeoxyglucose positron emission tomography with computed tomography (FDG PET-CT) scan showed metabolically active soft tissue mass in musculofascial plane of right lower thigh with FDG-avid right inguinal and external iliac lymph nodes, and multiple small bilateral lung nodules suspicious for metastases. In view of residual disease, she underwent wide local excision of the primary tumor along with right ilio-inguinal lymph node dissection. The tumor measured 8 × 5 × 5 cm, with all peripheral margins being negative. 10 out of 19 inguinal lymph nodes and 11 out of 22 pelvic lymph nodes showed metastatic tumor with extracapsular extension. On immunohistochemistry (IHC), tumor cells had a Ki-67 of 40%, and were positive for desmin, while being negative for SMA, S-100, CD34, CD99, Bcl2, MDM2, Desmin, H-caldesmon, cytokeratin, epithelial membrane antigen, Alk-1, HMB45, Melan-A, CK18, CK19, P63, ER, CD10, CK5/6, CK-HMW. She presented to our center at this point for further management and in view of metastatic disease, was advised doxorubicin-based chemotherapy. After discussion of the encouraging results from the phase 2 trial conducted by Tap et al. with the patient, the platelet derived growth factor receptor alpha antibody olaratumab was also added [2]. However, response assessment done after 4 cycles of doxorubicin and olaratumab showed progressive disease and she was then switched to gemcitabine and docetaxel regimen. She received 8 cycles of gemcitabine/docetaxel and had reduction in number and size of lung nodules, suggestive of partial response (Fig. 2a, b). Chemotherapy was stopped in view of unacceptable toxicity and she was kept on follow-up. After 3 months of treatment-free interval, she developed progressively worsening cough. A CT scan of the chest was done, which revealed disease progression (Fig. 2c, d). Biopsy blocks were reviewed for expression of programmed death ligand-1 (PD-L1) by IHC using the Ventana PD-L1 assay (SP263), which was positive, with a tumor cell score of 25 percent (Fig. 3). She was then started on pazopanib 800 mg daily along with anti-PD-1 antibody pembrolizumab pembrolizumab (200 mg every 3 weeks). She had grade 2 infusion reaction with second dose of pembrolizumab, grade 2 immune-related hypothyroidism and grade 2 hypertension. There was significant improvement in her cough within one month of therapy and response evaluation done after 3 months of therapy showed regression of lung metastases (Fig. 2e, f). Same combination was continued with no unmanageable adverse effects, and a repeat evaluation done after 9 months of treatment showed further disease regression (Fig. 2 g, h). Thus far, she has completed ten months of this combination therapy and continues to be in good general condition.

## Discussion and conclusions

Vascular endothelial growth factor (VEGF) has been implicated not only in tumor angiogenesis and metastases, but also plays a crucial role in maintaining an immunosuppressive tumor microenvironment. VEGF-VEGF receptor interactions have been shown to upregulate intratumoral regulatory T cells (Tregs), myeloid derived suppressor cells (MDSCs), and tumor-associated macrophages, while suppressing T cell function and interfering with differentiation and activation of dendritic cells [7]. VEGF receptor inhibitors, apart from their fundamental mechanism of inhibiting angiogenesis, have been shown to modulate the intratumoral cytokines and thereby infiltration by immune effector and suppressor cells [8]. The positive immunomodulatory activity of VEGF receptor tyrosine kinase inhibitors (TKIs) like sunitinib, pazopanib and axitinib in the tumor microenvironment underlies the proposition of combining them with immune checkpoint inhibitors to increase the therapeutic responsiveness of the latter [9]. Such combinations were initially explored in the setting of advanced renal cell carcinoma (RCC) and have yielded positive results [10]. In a single arm, phase 2 trial, VEGF TKI axitinib plus pembrolizumab was studied in 33 patients with advanced sarcoma who had progressed after or declined standard chemotherapy or targeted therapy [11]. Notwithstanding the limitations of cross-trial comparison, progression free survival at 6 months in this series compared favorably to the results obtained with monotherapy with either agent. However, objective responses were seen primarily in patients with alveolar soft part sarcoma (54.5%) while none of the 5 patients with UPS had an objective response. However, there have been concerns about the tolerability of such drug combinations. For instance, the combination of nivolumab and sunitinib for advanced RCC was associated with grade 3/4 adverse events in 82% and treatment discontinuation in 39% patients enrolled in one study [12]. In contrast to these findings, the combination of avelumab and axitinib was well tolerated and had comparable adverse events (including grade 3 and 4 adverse events) when compared to a VEGF TKI (sunitinib) alone in the JAVELIN Renal 101 trial [10].

As the patient was in significant distress due to persistent cough, and monotherapy with pazopanib and pembrolizumab is associated with modest objective response rates (6 and 23%, respectively), we considered combing the two agents in her case. Given the need to achieve a rapid tumor response, and evidence for the combination therapy resulting in meaningful results with acceptable toxicity, we were enticed to offer it to our patient after a discussion of the potential risks and benefits. She has tolerated the dual therapy well and has had no grade 3 or 4 adverse effects. Although each agent’s individual contribution to the good response observed in this patient can only be confirmed or refuted in a randomized trial, the improvement was most likely attributable to the immune checkpoint inhibitor since objective responses with single agent pazopanib are uncommon and typically not well-sustained [1]. Interestingly, the durable response achieved in our patient is in contrast to the poor response rate (only 1 minor response in 5 patients with UPS) seen in the trial by Wilky and colleagues [11]. The explanation may lie in the expression of PD-L1, or the fact that in contrast to axitinib, pazopanib inhibits multiple kinases that play a pivotal role in the tumor microenvironment and immune response [13].

In contrast to immunologically active tumors like melanoma, the tumor microenvironment in soft tissue sarcomas is considered immunologically quiescent. Sarcoma tumor cells have poor antigenicity and easily escape recognition by tumor infiltrating immune effector cells. However, undifferentiated pleomorphic sarcomas have high tumor mutation burden and may provide protein targets for immunotherapy. Extremity and trunk UPS biopsy samples have been shown to have an infiltrate of immune cells at baseline, and this infiltration by CD3, CD4, CD8 and FOXP3 positive cells is further enhanced by radiation therapy [14]. The possible mechanisms may include radiation induced increased antigenic expression, release of pro-inflammatory cytokines that recruit immune cells, promotion of antigen cross-presentation and increased death receptor expression in tumors [15].

In the SARC028 trial, only 3 out of 70 biopsy samples with advanced sarcoma were positive for PD-L1 expression at 1% threshold—all 3 cases were UPS. Although responses to pembrolizumab were seen even in the absence of PD-L1 expression, 2 evaluable patients with PD-L1 expressing tumors achieved partial response or complete response. The authors concluded that UPS fits the model of an inflamed tumor, with potential benefit from single agent anti-PD-1 antibodies. We may extrapolate these findings to suggest that patients with PD-L1 expressing UPS represent the most likely subset to benefit from anti-PD-1 antibodies. This biomarker analysis may allow better patient selection for consideration of immune checkpoint inhibitors. Outcomes from the expansion cohort of SARC 028 trial including total 40 patients with UPS have now been reported and have shown encouraging results. In these patients who failed one or more prior lines of therapy, pembrolizumab showed an overall response rate of 23% (9/40)—2 complete responses and 7 partial responses [16]. 75% of the responders had tumors positive for PD-L1 expression. The immune-related toxicities were predictable and manageable in most cases. Based on these positive outcomes, an open label, randomized phase 2 trial to assess the response to neoadjuvant nivolumab (anti-PD-1) alone or in combination with ipilimumab (anti-CTLA-4) with or without concurrent radiotherapy in patients with surgically resectable dedifferentiated liposarcoma and undifferentiated pleomorphic sarcoma has been designed and is currently recruiting [17]. The outcomes of this trial, expected in 2021, will further our understanding of the benefits of immunotherapy in these tumor subtypes. Considered together, these data indicate that undifferentiated pleomorphic sarcoma is an immunologically active subtype of soft tissue sarcoma, which is particularly amenable to immune checkpoint inhibitors. Clinical trials with inclusion of biomarkers such as PD-L1 expression may help identify patients most likely to benefit from immunotherapy.

Our case report adds to the currently scant literature on the results achieved from the combination of an immune checkpoint inhibitor and a VEGF TKI in advanced sarcomas, more specifically undifferentiated pleomorphic sarcoma. Considering that this dual therapy is feasible, well tolerated and can result in sustained response, these findings merit further evaluation in prospectively designed randomized clinical trials. Better understanding of the sarcoma microenvironment and response/resistance pathways is crucial to individualize therapy and improve patient outcomes.

## Data Availability

All data generated or analyzed during this study are included in this published article.

## Abbreviations

mSTS: Metastatic soft tissue sarcoma
US FDA: United States Food and Drug Administration
ASCO: American Society of Clinical Oncology
LMS: Leiomyosarcoma
UPS: Undifferentiated pleomorphic sarcoma
anti-PD1: Anti-programmed death protein-1
FNCLCC: Fédération Nationale des Centres de Lutte Contre Le Cancer
FDG PET-CT: 18-fluorodeoxyglucose positron emission tomography with computed tomography
IHC: Immunohistochemistry
PD-L1: Programmed death ligand-1
VEGF: Vascular endothelial growth factor
Tregs: Regulatory T cells
MDSCs: Myeloid derived suppressor cells
TKI: Tyrosine kinase inhibitor
RCC: Renal cell carcinoma

## References

[1] van der Graaf WT, Blay J-Y, Chawla SP, Kim D-W, Bui-Nguyen B, Casali PG (2012). Pazopanib for metastatic soft-tissue sarcoma (PALETTE): a randomised, double-blind, placebo-controlled phase 3 trial. Lancet.

[2] Tap WD, Jones RL, Tine BAV, Chmielowski B, Elias AD, Adkins D (2016). Olaratumab and doxorubicin versus doxorubicin alone for treatment of soft-tissue sarcoma: an open-label phase 1b and randomised phase 2 trial. Lancet.

[3] Tap WD, Wagner AJ, Papai Z, Ganjoo KN, Yen C-C, Schoffski P (2019). Announce: a randomized, placebo (PBO)-controlled double-blind, phase (Ph) III trial of doxorubicin (dox) + olaratumab versus dox + PBO in patients (pts) with advanced soft tissue sarcomas (STS). J Clin Oncol.

[4] Tawbi HA, Burgess M, Bolejack V, Tine BAV, Schuetze SM, Hu J (2017). Pembrolizumab in advanced soft-tissue sarcoma and bone sarcoma (SARC028): a multicentre, two-cohort, single-arm, open-label, phase 2 trial. Lancet Oncol.

[5] George S, Barysauskas CM, Solomon S, Tahlil K, Malley R, Hohos M (2016). Phase 2 study of nivolumab in metastatic leiomyosarcoma of the uterus. J Clin Oncol.

[6] Toulmonde M, Penel N, Adam J, Chevreau C, Blay J-Y, Cesne AL (2018). Use of PD-1 targeting, macrophage infiltration, and IDO pathway activation in sarcomas: a phase 2 clinical trial. JAMA Oncol.

[7] Yang J, Yan J, Liu B. Targeting VEGF/VEGFR to modulate antitumor immunity. Front Immunol [Internet]. 2018 May 3–9.https://www.ncbi.nlm.nih.gov/pmc/articles/PMC5943566/. Accessed 25 Jun 2020 10.3389/fimmu.2018.00978 PMC5943566 29774034

[8] Roland CL, Lynn KD, Toombs JE, Dineen SP, Udugamasooriya DG, Brekken RA (2009). Cytokine levels correlate with immune cell infiltration after anti-VEGF therapy in preclinical mouse models of breast cancer. PLoS ONE.

[9] Fukumura D, Kloepper J, Amoozgar Z, Duda DG, Jain RK (2018). Enhancing cancer immunotherapy using antiangiogenics: opportunities and challenges. Nat Rev Clin Oncol.

[10] Motzer RJ, Penkov K, Haanen J, Rini B, Albiges L, Campbell MT (2019). Avelumab plus axitinib versus sunitinib for advanced renal-cell carcinoma. N Engl J Med.

[11] Wilky BA, Trucco MM, Subhawong TK, Florou V, Park W, Kwon D (2019). Axitinib plus pembrolizumab in patients with advanced sarcomas including alveolar soft-part sarcoma: a single-centre, single-arm, phase 2 trial. Lancet Oncol.

[12] Amin A, Plimack ER, Ernstoff MS, Lewis LD, Bauer TM, McDermott DF (2018). Safety and efficacy of nivolumab in combination with sunitinib or pazopanib in advanced or metastatic renal cell carcinoma: the CheckMate 016 study. J Immunother Cancer.

[13] van Geel RMJM, Beijnen JH, Schellens JHM (2012). Concise drug review: pazopanib and axitinib. Oncologist.

[14] Keung EZ, Tsai J-W, Ali AM, Cormier JN, Bishop AJ, Guadagnolo BA (2018). Analysis of the immune infiltrate in undifferentiated pleomorphic sarcoma of the extremity and trunk in response to radiotherapy: rationale for combination neoadjuvant immune checkpoint inhibition and radiotherapy. OncoImmunology.

[15] Kalbasi A, June CH, Haas N, Vapiwala N (2013). Radiation and immunotherapy: a synergistic combination. J Clin Invest.

[16] Burgess MA, Bolejack V, Schuetze S, Van Tine BA, Attia S, Riedel RF (2019). Clinical activity of pembrolizumab (P) in undifferentiated pleomorphic sarcoma (UPS) and dedifferentiated/pleomorphic liposarcoma (LPS): final results of SARC028 expansion cohorts. J Clin Oncol.

[17] Keung EZ, Lazar AJ, Torres KE, Wang W-L, Cormier JN, Ashleigh Guadagnolo B (2018). Phase II study of neoadjuvant checkpoint blockade in patients with surgically resectable undifferentiated pleomorphic sarcoma and dedifferentiated liposarcoma. BMC Cancer.

